# Medical Scholarships Linked to Mandatory Service: The Nepal Experience

**DOI:** 10.3389/fpubh.2020.546382

**Published:** 2020-10-26

**Authors:** Agya Mahat, Mark Zimmerman, Rabina Shakya, Robert B. Gerzoff

**Affiliations:** ^1^Nick Simons Institute, Lalitpur, Nepal; ^2^Retired, Atlanta, GA, United States

**Keywords:** medical education, medical scholarships, rural physicians, private sector, health policy

## Abstract

**Introduction:** Nepal has one of the world's lowest physician to population ratios, with a critical shortage of rural physicians. The Nepal Government uses the private sector to address this shortage of rural physicians. All private medical colleges must offer total scholarships, free of cost, to a proportion of their annual MBBS student intake. These scholarships come with a compulsory two-year service contract, which must be completed at public hospitals post-graduation. The mandatory service requirement was implemented in 2005/2006 and this paper evaluates the first decade of this scholarship program, with particular attention to the mandatory service requirement.

**Methods:** We collected data on MBBS scholarship awardees from the Scholarship Section at the Ministry of Education, Department of Health Services, and the Ministry of Health and evaluated trends, service completion, and location.

**Results:** Initially, because of poor monitoring, the mandatory service completion rate was low. Rates increased to 74–98% when strict rules tied service completion certificates to obtaining medical registration. In the past 4 years, three cohorts of scholarship doctors who completed their service requirements served 78% of their service-days in rural hospitals (primary healthcare centers and district hospitals). Yet, geographic inequities in physician distribution persist. Only 51% of district hospitals had at least one scholarship doctor, 31% of the district hospitals had more than 1.5 scholarship doctors, while 7% had none. The district hospitals in the Central region, which includes the capital city, had twice the number of scholarship doctors compared to the Mid-western region, which includes some of the country's most remote areas.

**Conclusion:** The scholarship program has partially succeeded in reducing the physician shortage in Nepal's rural hospitals. To address the remaining inequities in physician distribution, efficient management systems, appropriate medical training, and support for rural practice are vital.

## Introduction

The World Health Organization recommends medical scholarships “with enforceable agreements of return of service in rural or remote areas to increase the recruitment of health workers in those areas” (1). Although conditions, incentives, and duration of mandatory service differ, a compulsory service strategy has been used in over 70 countries to recruit physicians to underserved areas (2). For example, compulsory service strategies ensured that all districts in Mozambique and all municipalities in Puerto Rico had at least one doctor (2, 3), staffing improved in rural hospitals of South Africa (4), and physicians were more equitably distributed Thailand's district hospitals (5). However, compulsory service in five Indian states did not effectively place doctors in underserved areas (6).

Nepal is a low-income country with one of the world's lowest physician to population ratios of 2.1: 10,000 (7). This low ratio is coupled with inequities in physician distribution. Eighty-five percent of specialists and 56% of MBBS (Bachelors in Medicine Bachelors in Surgery) public sector doctors work in the Central region that includes the capital, Kathmandu (8).

The recruitment and retention of physicians in rural areas, where 83% of the population resides, has been a persistent challenge. In 2013, over half of the positions for doctors in rural public hospitals (77% of primary healthcare centers and 53% of district hospitals) were reported to be vacant (9). National health policies since 1991 have aimed to increase access of the rural population to a doctor through strategies including scaling up the production of different categories of health workers through the private sector, increasing the quality of pre-service education, and encouraging the deployment of graduates to rural areas (10–13).

This paper reviews government policy to use training scholarships to leverage the private medical education sector and improve physician placement in rural areas through mandatory 2-year service in public hospitals. It highlights that the successful enforcement of such a policy is dependent on the government's capacity to fund, regulate, coordinate, plan and provide adequate support and training for physicians.

In the early 1990s, the Nepal government allowed the private sector to offer medical education within the country. Consequently, ~2,000 new MBBS doctors now graduate from Nepal's medical colleges every year. The private sector produces the largest number of physicians. Seventeen of the 20 medical colleges that currently offer an MBBS program are private. A 5.5-year MBBS program at a private institution costs about $40,000 USD. At a public institution, it can be as little as $2,500 USD.

In Nepal, the government requires that all private medical colleges offer complete scholarships, which offer free admission and tuition to a proportion of their annual student intake (10% for Nepali-owned, 20% for foreign-owned for-profit institutions). The number of scholarships differs each year depending on the number of students approved for intake by the Nepal Medical Council (NMC). These scholarships include a mandatory, post-graduation, 2-year work contract in government health facilities. These private institutions fund medical education, and the government pays the recipients' salaries during the mandatory service period.

The medical scholarship funding from the private sector has relieved the government of expenses otherwise required to produce doctors for its rural hospitals. However, the mandatory service requirement has only been enforced since 2005 (for the scholarship cohort of 2000). Until then, the government did not have the resources to pay the salaries of the scholarship doctors after graduation.

### The MBBS Scholarship Program

The Ministry of Education (MoE) selects the scholarship awardees based on their performance in a competitive exam. This complies with the government directive, *Scholarship Rules 2060 (2003) (2002/2003 AD)* (14). According to these rules, 55% of the scholarship awards are open category, meaning that they are entirely based on merit while 45% of the awards are reserved for under-represented groups such as women, indigenous, socially/economically excluded groups, and people from remote areas (Table 1).

**Table 1:** Reservation category for Ministry of Education scholarships [adapted from Government of Nepal (14)].

**Reserved category**	**Proportion of reserved category**	**Proportion of total scholarship**
Women	33%	14.85%
*Janajatis* (indigenous group)	27%	12.15%
Economically or socially excluded	25%	11.25%
Dalit	9%	4.05%
Citizens from remote areas (*Accham, Kalikot, Jajarkot, Jumla, Dolpa, Bajhang, Bajura, Mugu, and Humla* districts)	4%	1.8%
Disabled	2%	0.9%
Total	100%	45%

Under-represented groups can compete in both open and reserved categories. If a candidate from a reserved-population group who graduated from a community school does not pass the MoE exam, someone from the same population group graduating from other types of schools (e.g., private, non-profit, missionary, or public-private.) becomes eligible for the scholarship award. If candidates of the under-represented groups fail to pass the minimum requirements to fill the quota for the reserved category, the scholarships are awarded to candidates in the open category.

Within 3 months of graduation from medical college, scholarship doctors are required to report to the MoE and then to the Department of Health Services (DoHS) under the Ministry of Health (MoH) for posting at a public hospital. Late reporting results in a financial penalty. The DoHS places scholarship doctors in public hospitals based on vacant hospital positions at the time of reporting. The scholarship doctors are posted to a health facility for 2 years unless they have received a transfer letter. They may request a transfer themselves or be transferred by the DoHS to another hospital at any time.

During their mandatory service period, scholarship doctors are eligible to apply for permanent government service positions. If accepted in the Ministry of Health or any of the public security agencies (Nepal Army, Nepal Police, and Nepal Armed Police Force), they can complete their mandatory service period as permanent government employees.

After 2 years of service, DoHS issues the scholarship doctors a certificate of service completion. Scholarship doctors who do not complete their service requirements are required to pay the government a financial penalty. If they do not complete their service nor pay the financial penalty, the NMC will not issue them a permanent registration nor will MoE issue a *Letter of no objection*. The NMC registration is required to practice medicine in Nepal, and the *Letter of no objection* is required for immigration clearance to leave the country on a student visa.

### Nepal's Public Health Care System

**Figure 1:**
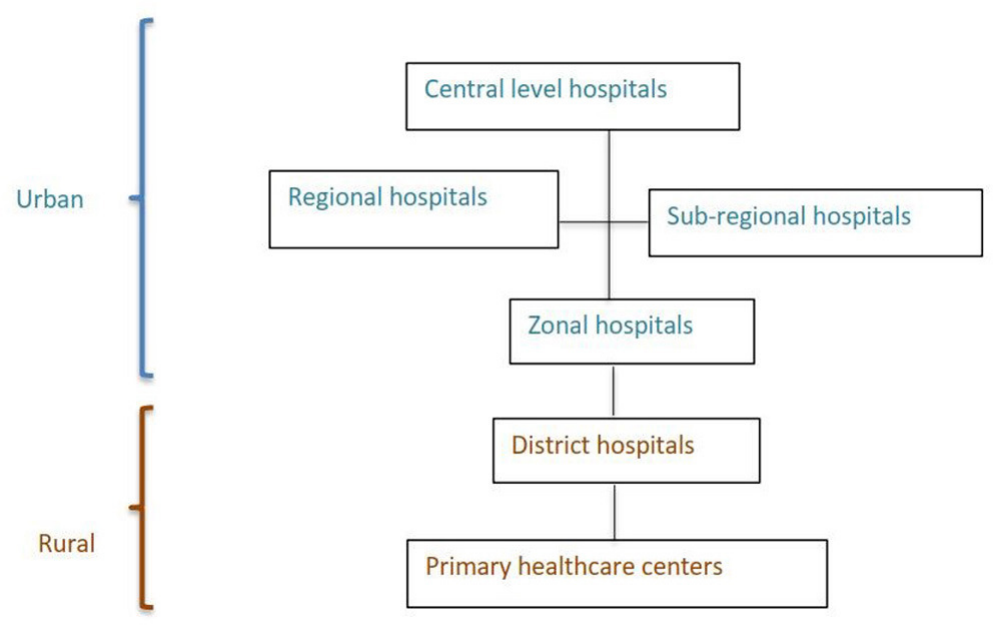
Nepal's hospital structure.

Nepal's healthcare system follows a hierarchical hospital referral structure (Figure 1). Primary healthcare centers (PHCs) and District hospitals provide healthcare services to the rural population. District hospitals provide lifesaving emergency surgical services. Zonal, Sub-Regional, and Regional hospitals receive referral cases from their respective areas. The Central hospitals located in Kathmandu valley provide tertiary care and super-specialized services.

All districts have at least one district hospital or a referral hospital (zonal, sub-regional, regional, or central hospital). Sixty-three out of 75 districts have a district hospital. Some districts have more than one district hospital because a PHC was upgraded.

### Objective

This paper evaluated the first decade of Nepal's MBBS scholarship program. Specifically, it sought to answer the following:

- In the past 10 years, how many citizens were awarded MBBS scholarships to Nepal's private colleges?
- What percentage of the scholarship awardees completed their mandatory service?
- Where did the awardees serve their mandatory service and for how long?

## Methods

We collected the number and place of MBBS scholarship awards, the mandatory post-graduate service duration, and the completion status of the scholarship doctors. We merged data obtained from the DoHS, MoH, and the Scholarship Section of the MoE. Our study was limited to awardees at Nepal's private medical colleges.

We examined scholarship trends, service completion rates and service location.

Data on the category selected for the scholarship were often missing. The data on sub-categories (under the reservation category) for scholarship doctors was not available. Mandatory service data was also often incomplete. While for some years the scholarship doctors' documents were complete, other years did not have data on the location and duration of service during the bonded contract period. Furthermore, data on the location and duration of scholarship doctors' service were available only for the most recent three cohorts that completed their mandatory service (scholarship cohorts of 2005–2007). Therefore, we limited our service completion data analysis to these three cohorts only. The duration of service was calculated from the start date and end date per facility in the service completion certificates.

## Results

**Figure 2:**
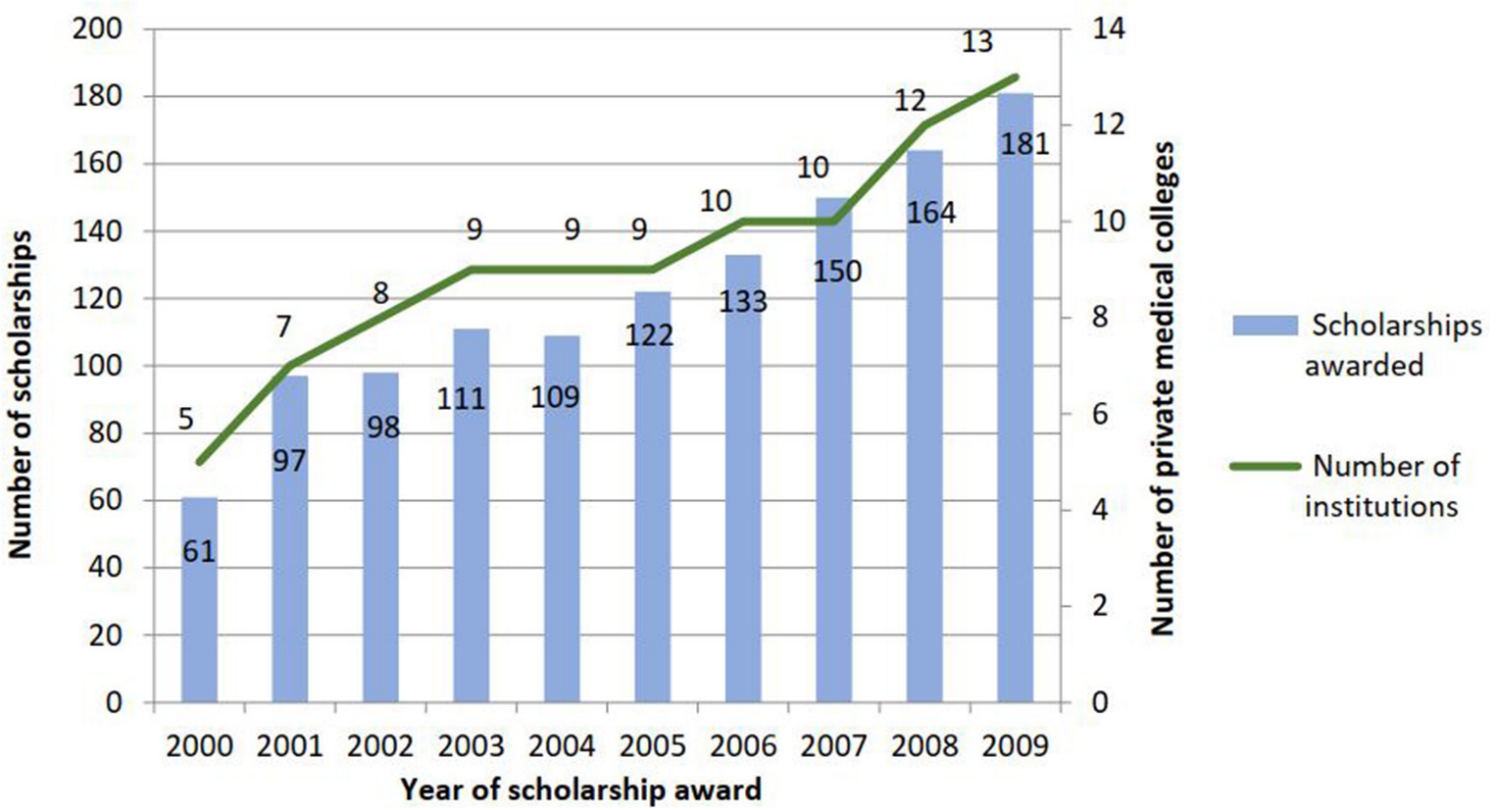
Number of private medical colleges and scholarships awarded (2000–2009).

From 2000 to 2009, the MoE awarded 1,226 MBBS scholarships (Figure 2). The number of scholarships increased an average of 14% each year. The largest increase was in the first year (59% between scholarship cohorts 2000 and 2001) after which the number of scholarships increased by 8% per year. In 2009, 181 citizens were awarded MBBS scholarships in 13 private institutions compared to 60 scholarships in 5 institutions in 2000. This is a 197% increase in the number of scholarships and a 160% increase in the number of private medical colleges in 10 years.

Since the introduction of the reservation category in 2003 up until 2009, 58% of the scholarship awardees have been from the open category and 36% from the reservation category (with 6% missing data) (Table 2). This is less than the target ratio of 55:45. In 2009, 43% of the scholarships were awarded to the reserved category.

**Table 2:** Categories of medical scholarship recipients (2000–2009).

**Scholarship year**	**Open category**	**Reserved** **category**	**Missing data**	**Total**
2000	61(100%)	–		61
2001	105 (100%)	–		105
2002	98 (100%)	–		98
**Total (2000** **−2002)**	**264 (100%)**			**264**
2003	68 (61%)	39 (35%)	4 (4%)	111
2004	65 (60%)	44 (40%)	0	109
2005	76 (62%)	44 (36%)	2 (2%)	122
2006	75 (56%)	45 (34%)	13 (10%)	133
2007	83 (55%)	44 (29%)	23 (15%)	150
2008	94 (57%)	59 (36%)	11 (7%)	164
2009	98 (54%)	77 (43%)	6 (3%)	181
**Total (2003–2009)**	**559 (58%)**	**352 (36%)**	**59 (6%)**	**970**

The vast majority (81%) of the scholarship recipients are male (Table 3). Before the reservation category for under-represented groups, only 3–11% of scholarship recipients were women. Since 2003, because they can also compete under both open and reserved categories, the proportion of female scholarship awardees has increased and is consistently more than the reserved 15% of the total scholarships. In 2004, 30% of the scholarships were awarded to women.

**Table 3:** Sex disaggregation of scholarship awardees in private medical colleges (2000–2009).

**Scholarship cohort**	**Female (%)**	**Male (%)**	**Remarks**
2000	2 (3%)	59 (97%)	
2001	6 (6%)	91 (94%)	
2002	11 (11%)	87 (89%)	
2003	27 (24%)	84 (76%)	Reservation category enforced (14.85% of total scholarships for women)
2004	33 (30%)	76 (70%)	
2005	31 (25%)	91 (75%)	
2006	33 (25%)	100 (75%)	
2007	32 (21%)	118 (79%)	
2008	26 (16%)	138 (84%)	
2009	37 (20%)	144 (80%)	
**Total**	**238 (19%)**	**988 (81%)**	

**Figure 3:**
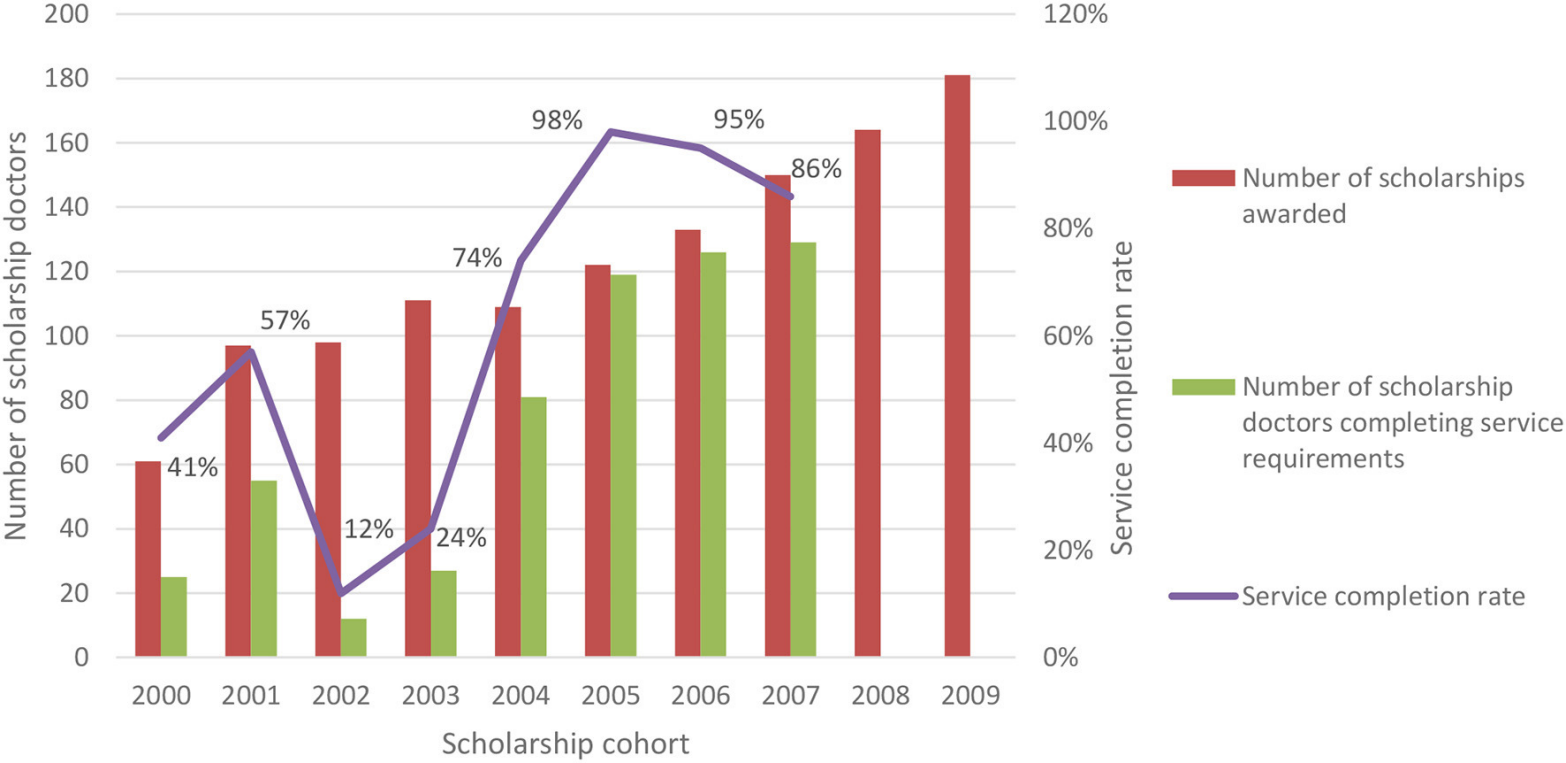
Number of scholarships awarded and service completion rate of the scholarship cohorts.

The scholarship awardees' service completion rate has increased over the past decade (Figure 3). Only 12–57% of the first four cohorts of scholarship awardees, between 2000 and 2003, completed their service, but 74% of the fifth cohort in 2004 completed their mandatory service. The service completion rate for the next three cohorts between 2005 and 2007 then ranged from 86 to 98%. The scholarship cohorts of 2008 and 2009 were in service at the time we collected data.

Over half of the scholarship doctors served in the same health facility for 2 years, 28% in two facilities, and 15% in three (Table 4). Among the 6% of doctors who served in more than 3 health facilities, most are those who, during their mandatory service period, joined the Ministry of Health as permanent employees.

**Table 4:** Number of healthcare facilities in which the scholarship doctors were posted (scholarship cohorts 2005−2007).

**Number of healthcare** **facilities served**	**Number of doctors**	**Percent**
1	192	51%
2	104	28%
3	54	15%
4	19	5%
5	4	1%
**Total**	**373**	**100%**

We calculated the scholarship doctors' service-days from their DoHS certificate of service completion. Of the 374 doctors of the three cohorts who completed their service, details were available for all but one. The 373 doctors provided 271,067 service-days (Table 5) an average of 726.7 days of service (1.99 years) per scholarship doctor.

**Table 5:** Service days of scholarship doctors in different health facility types.

**Types of health facility**	**Number of HFs served (total HFs)**	**Service-days**	**Percent of service days in different health facility types**
Ministry of Health		3,525	1%
Central Hospital	3	3,533	1%
Regional Hospital^*^	3 (3)	7,466	3%
Sub Regional Hospital	3 (3)	8,622	3%
Zonal Hospital	11 (11)	33,752	12%
District (public) Health Office		1,716	1%
District (level) Hospital	66 (71)	124,726	46%
Primary Healthcare Center	118 (205)	87,727	32%
**Total**		**271,067**	**99%**

* *Includes Regional Tuberculosis center*.

Considered together, the scholarship doctors completed three-quarters of their service-days in district hospitals (46%) and PHCs (32%), which serve rural populations. They completed 2% of the service-days in zonal hospitals, 3% each at sub-regional and regional hospitals, and the remainder at central hospitals of public security agencies, Ministry of Health, and District Health Offices.

**Figure 4:**
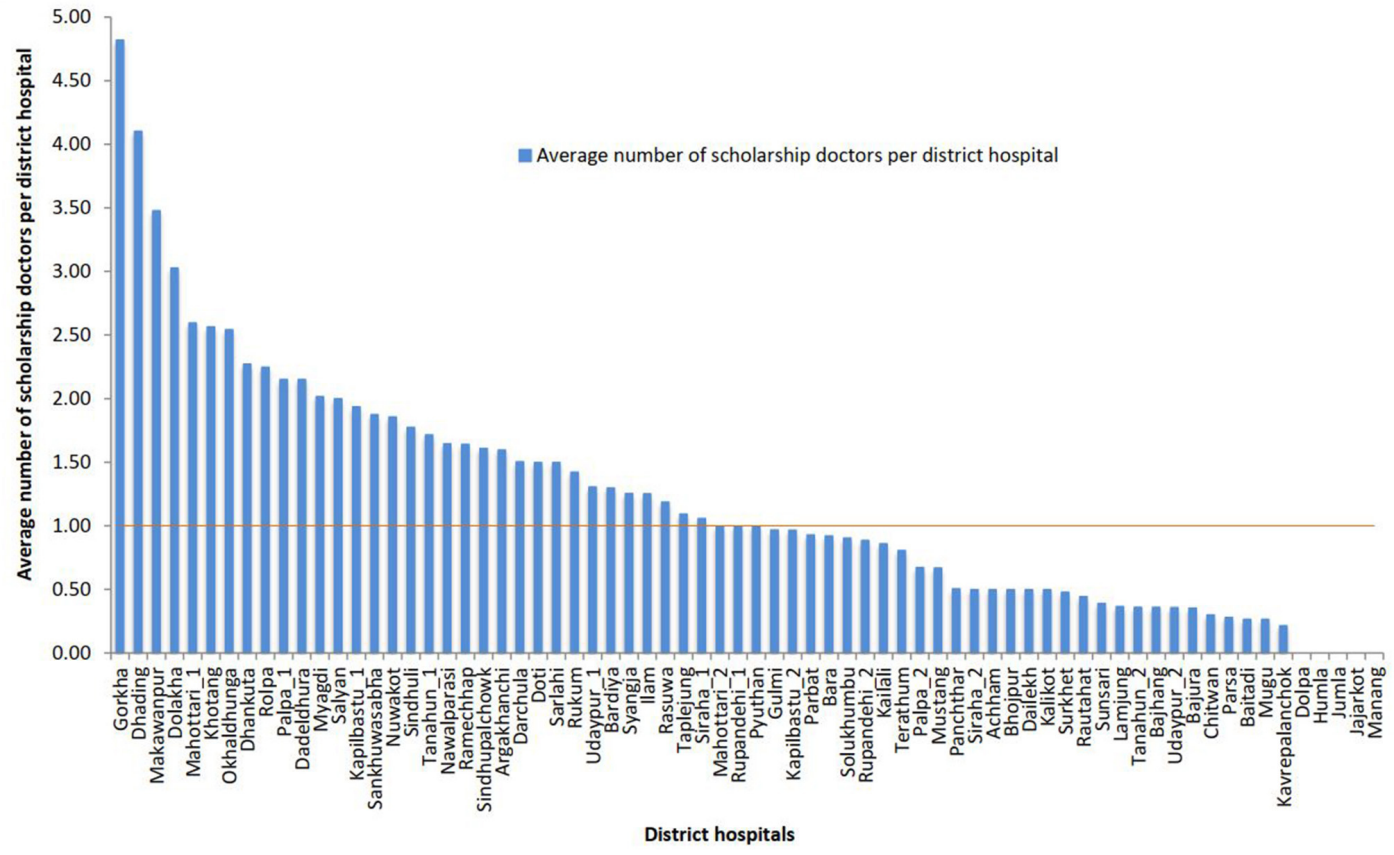
Average number of scholarship doctors per district hospital in the past 4 years.

Combining the last three cohorts that completed the mandatory service requirements, Figure 4 shows the average number of scholarship doctors in district hospitals. In total, the doctors provided 124,726 service-days at district hospitals in the past 4 years. Had they been equitably distributed across the 70 district hospitals, each hospital would have had 1.22^1^ scholarship doctors, i.e., at least one scholarship doctor would be present in all district hospitals.

Between 2012 and 2015 (the mandatory service period of the scholarship cohorts 2005–2007), up to five scholarship doctors worked in a district hospital at a time. Thirty-six district hospitals (51%) had one or more scholarship doctors while 34 hospitals (49%) had less than one. Twenty-two district hospitals (31%) had more than 1.5 doctors, of which 3 hospitals (4%) had more than 3 scholarship doctors. In contrast, no scholarship doctor served in five district hospitals (7%).

**Figure 5:**
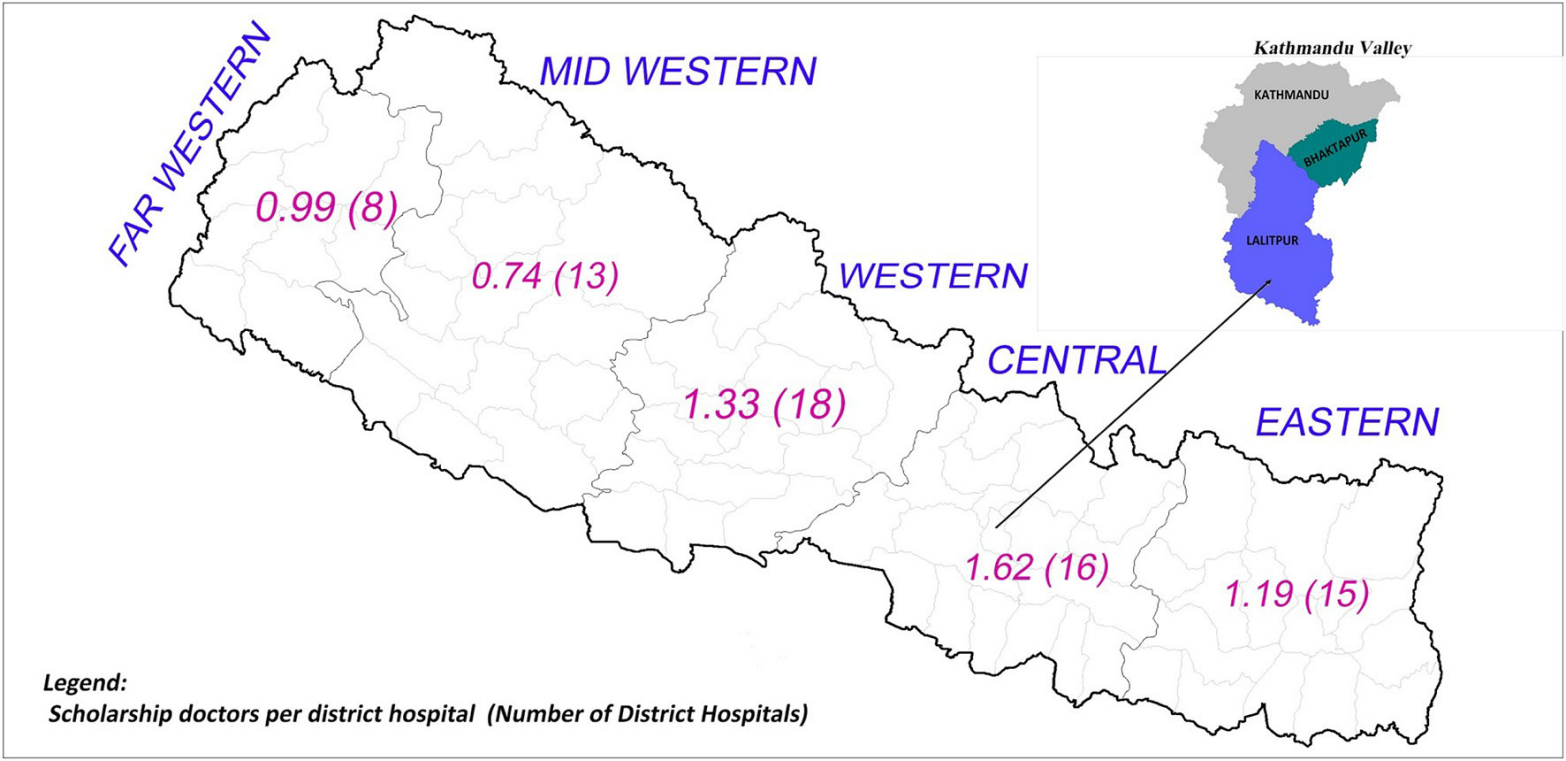
Average number of scholarship doctors per district hospital across development regions in the past 4 years.

The number of scholarship doctors in district hospitals varied by the development region (Figure 5). The Central, Western, and Eastern development regions had more than one scholarship doctor per hospital while the Mid-western and Far-western regions had fewer. Compared to the Mid-western region, hospitals in the Central region had twice the number of scholarship doctors (1.6 vs. 0.74).

## Discussion

In the early years of the mandatory service enforcement in Nepal, the scholarship doctors' service completion rate was low (scholarship cohorts of 2000–2003) because of weak program monitoring. To resolve this, the government enforced new rules that tied the service completion certificate to the *MoE Letter of No Objection* and the NMC registration. These rules required the doctors to present official letters and attendance sheets from the hospitals as evidence of their service. Consequently, the service completion rate increased from 24 to 74%. It also provided detailed data to the agencies at the MoH and MoE on the duration of service at each hospital. In the last three scholarship cohorts, the annual mandatory service completion rate has consistently been over 85%.

Government regulations appear to ensure a high service completion rate for those intending to practice medicine in Nepal. However, loopholes exist for defaulters who leave the country on a non-student visa or who quit the medical practice. Vietnam, Mongolia, and Ethiopia have used stricter measures such as withholding degrees until mandatory service is completed (2).

The proportion of medical scholarships selected entirely on merit dropped from 100% in 2001 to 54% in 2009. During this period, the proportion of women increased from 3% in 2001 to 30% in 2004. The reservation category for scholarships has given people from under-represented and excluded population groups the opportunity for medical education free of cost which would otherwise not be possible.

Nepal's scholarship program provides a regular supply of physicians for rural public hospitals. Of the 1,447 approved positions for doctors in the public sector in 2011, 335 (23%) were for those coming through the scholarship program (15). Before enforcing mandatory service obligations, doctors were less likely to work in rural areas. For instance, of the first 22 cohorts of Nepal's oldest medical college, only 27% of MBBS graduates worked outside the Kathmandu valley (16). In the public sector, only 32% of the generalist medical doctors work in rural areas (8). In contrast, the past three cohorts of scholarship doctors spent 98% of their first 2 years of practice outside of the Kathmandu valley and 78% in district hospitals and primary healthcare centers.

Despite these promising figures, geographic inequities in physician distribution remain. In the past 4 years, an overwhelming majority of scholarship doctors served in district hospitals in the Central, Western, and Eastern development regions. The Mid-western and Far-western regions, where the scholarship doctors served the least, are furthest from Kathmandu and include some of the most remote areas of the country. These findings are similar to the distribution of medical specialists, 85% of whom are based in the Central region (543), with only 5% in the Mid-western (*n* = 22) and Far-western (*n* = 4) regions. In the private sector, 68% of the specialists (*n* = 894) work in the Central region compared to 4% in the Mid-western (*n* = 29) and Far-western (*n* = 19) regions (8).

The scholarship program has been unable to place doctors in some areas with the greatest need. Although over three-quarters of the service-days of the scholarship doctors were spent in rural areas (either district hospitals or primary healthcare centers), their distribution varied according to their proximity to urban centers. The Mid-western region has the highest human poverty index in the country (17). Half of the children under five are mal-nourished (17) and the diarrheal mortality rate is twice the national average (18). The average life expectancy in the five districts where no scholarship doctors were posted (Dolpa, Humla, Jumla, Jajarkot, and Manang) ranges from 61 to 66 years, substantially below the national average of 69 years (13). The US National Health Service Corps, a program that provides loan repayment or medical scholarships for primary care service in underserved areas encountered similar difficulties. Areas with worse population health measures were less likely to benefit from program physician placements (19, 20). The inequity in the geographic distribution of doctors can be attributed to several factors.

### Program Management

Several issues concerned with the management of the program contribute to the geographic inequity. The main offices responsible for the MBBS scholarship program, the MoE and the DoHS do not coordinate or communicate about scholarship doctors. Data on the selected category of the scholarship doctors, the expected number of doctors from the mandatory service program, and the availability of physicians in each government facility under different types of contracts at any given time are not available in one platform. Hence, planning for appropriate positions is absent.

A transparent and accessible system is required to assign scholarship doctors to different hospitals. For example, in Norway, each graduate is randomly given a number and within 6 h of receiving the number, the graduates are called in numerical order to choose from among the available places (2). In Nepal, since the placement of scholarship doctors is at the discretion of DoHS officials, the doctors can exert influence, avoid the most remote postings, and be assigned to locations near big cities. The *Implementation Guidelines for Nepal Government Scholarship Recipient Doctors and Health-workers 2071* (2015) attempts to prevent doctors concentrating in certain hospitals by specifying the maximum number of scholarship doctors that can be posted at a health facility (21).

The timing and duration of a posting are also important. Although the scholarship doctors should report for service at the DoHS within 3 months of graduation, they are still eligible for posting beyond this period after paying a small financial penalty. This allows them to delay starting work until positions near urban centers become vacant. For the past 4 years, 21% of the scholarship doctors were posted for an average of less than a year in one healthcare facility. This shows the instability of staffing in public hospitals.

Finally, MoE tracks defaulters only after someone from the general public files a petition against a defaulter. The defaulter can still choose to serve the mandatory period after payment of a late fee instead of the larger financial penalty for breaking the terms of the contract. Regular tracking of scholarship recipients is necessary to ensure scholarship doctors fulfill their service obligations within a specified period or pay a bigger penalty. Local governments and/or communities could be utilized for this tracking (19).

### Medical Training

There is no evaluation of the scholarship doctors' capacity and motivation to work in Nepal's remote. Such evaluations are important because often, the for-profit private medical colleges are designed to serve the global market rather than the local population's primary health care needs (22).

For new graduates, Nepal's remote public hospitals are extremely challenging workplaces. They often have a high volume of patients, are understaffed, face shortages of hospital equipment and supplies, laboratory, and radiological diagnostic support are limited or absent, and the sites have poor communication facilities. Supportive supervision may be poor or absent. Although the government has implemented a telemedicine program in 30 district hospitals so specialists can provide support in diagnosis and management, it is yet to be fully utilized because of challenges such as inconsistent power supply, inadequate information technology capacity, infrastructure challenges, and funding shortages (23).

Another solution might be to follow the experiences of the scholarship system in South Africa, where the internship period for doctors has increased to 2 years to prepare them for independent practice in district hospitals (24). The first year of mandatory service could be based in larger hospitals to build the capacity of recent graduates' to work in remote areas the second year.

### Additional Support

Scholarship doctors are paid a graded salary. Additional rural financial incentives are provided for those working in rural and remote areas. However, financial incentives are not sufficient to ensure quality healthcare services. For Nepal's scholarship doctors, there are no additional advantages to serving in rural postings except for being eligible to apply for openings at government-owned institutions during the mandatory service period.

Mandatory rural service programs work best when combined with additional support such as comfortable housing, central personnel management, security, and a supportive working environment (1, 25, 26). The current program has not considered these factors even as the government enforced mandatory service for MBBS from public medical colleges from 2015 and for all specialists under government scholarships since 2018.

## Limitations

This study depended entirely on the data available at the DoHS and MoE, as no other source was available. MBBS doctors working in public hospitals are recruited through permanent positions at MoH, temporary contracts at some hospitals, and through the MBBS scholarship program. All these doctors are managed through different human resource management systems within different divisions and departments of MoH. We focused solely on the doctors recruited through the MBBS scholarship program for private medical colleges. Without data on non-scholarship doctors, we cannot say what portion of the public sector MBBS positions were filled through this program.

Data on defaulters was not available. The penalty amount is deposited in the Internal Revenue Department, and this information is not recorded in the relevant agencies within the Ministries of Education or Health. We cannot say if those graduates not completing the mandatory service requirements paid the financial penalty, if they dropped out of medical college, or avoided the rural postings after graduation.

The scholarship doctors' service-days have been calculated from their DoHS certificate of service completion. These counts may include holidays and entitled leave. The scholarship doctors are allowed 30 days of leave that cannot be taken together, but we do not have data on how much of the entitled leave was used. Therefore, one must interpret the service-days results with caution.

The inconsistency in data completeness (e.g., details on the category of scholarship, the background of doctors, details on service, data on other graduates) did not allow for a rigorous analysis on the association of the scholarship doctors characteristics with the likelihood of rural service and limited our analysis to service completion status of three cohorts only.

Finally, this study does not consider the quality of medical education, working conditions in rural hospitals, the career paths of the scholarship doctors, and stakeholders' perspectives (e.g., scholarship doctors, hospital managers/supervisors, patients, MoE, and MoH representatives). These are critical factors that determine program effectiveness and must be considered when improvement recommendations are developed.

## Conclusion and Recommendations

Nepal's MBBS scholarship program has provided considerable medical education opportunities to the citizens including under-represented groups and communities without using government funds. The program has partially succeeded in addressing the rural physician shortage.

Low- and middle-income countries struggling to place doctors in rural hospitals can learn from Nepal's experience leveraging the private sector to fill the human resource for health gaps in rural public hospitals. To reap the full benefit of the program, along with mandatory rural service contract, the emphasis must also be on (a) the equitable distribution of MBBS physicians during their mandatory service period, (b) the creation of favorable working conditions to deliver high-quality service, and (c) provision of appropriate physician training for practice at remote health facilities to achieve the objectives of the MBBS Scholarship program.

Considering this, we make the following recommendations:

1. *Improve coordination between relevant agencies* to ensure physician production and postings are well-planned, medical training is aligned with population needs, infrastructure and logistical support are available in rural health facilities, defaulters are actively tracked and penalized, and the program meets its objectives through a consolidated health workforce department that hosts accurate data on the health workforce.
2. *Establish information systems* under different ministries that record complete information and are compatible and ensure access, input, and import relevant digital data for regular monitoring, analysis, and evaluation to support planning.
3. *Enforce mandatory rural service contracts* for physicians trained under government scholarships or subsidies. Medical degrees and/or medical council registrations should be conditional upon service fulfillment.
4. *Utilize the private sector for government scholarships* ensuring that under-represented groups and rural populations are included in the scholarships.
5. *Ensure that the quality of medical education* and the training curricula meets the health needs of the local population.
6. *Create a systematic and transparent process for posting scholarship doctors* based on local needs, that avoids cherry-picking postings and taking into account the background, availability, and to some extent, graduates' preferences.
7. *Provide adequate support and bundled incentives to rural physicians* such as comfortable housing, security, communication support including operational telemedicine services, supportive supervision, and mentorship from senior role models in government hospitals, opportunities for continuing medical education, peer support, reasonable financial bonus, and opportunities for training and promotions.

## Author Contributions

AM conceived the study and led data collection. AM, RBG, RS, and MZ contributed to data analysis. AM and RBG contributed to writing the manuscript. RS provided coordination and administrative support. MZ supervised the study. All authors contributed to the article and approved the submitted version.

## Conflict of Interest

The authors declare that the research was conducted in the absence of any commercial or financial relationships that could be construed as a potential conflict of interest.

## Footnotes

^1^Number of scholarship doctor per district hospital = (Total number of service-days in district hospital)/(number of district hospitals^*^number of years^*^number of days in a year).

## Abbreviations

DoHS: Department of Health Services
HF: Health facility
MoE: Ministry of Education
MoH: Ministry of Health
MBBS: Bachelor in Medicine Bachelor in Surgery
NMC: Nepal Medical Council
PHC: Primary healthcare center.
